# Gaze training supports self-organization of movement coordination in children with developmental coordination disorder

**DOI:** 10.1038/s41598-018-38204-z

**Published:** 2019-02-08

**Authors:** Piotr Słowiński, Harun Baldemir, Greg Wood, Omid Alizadehkhaiyat, Ginny Coyles, Samuel Vine, Genevieve Williams, Krasimira Tsaneva-Atanasova, Mark Wilson

**Affiliations:** 1 College of Engineering, Mathematics, and Physical Sciences, University of Exeter, Exeter, EX4 4QJ UK; 2 Living Systems Institute, University of Exeter, Exeter, EX4 4QJ UK; 3 Translational Research Exchange @ Exeter, University of Exeter, Exeter, EX4 4QJ UK; 4 Department of Sport and Exercise Sciences, Manchester Metropolitan University, Manchester, M1 5GD UK; 5 School of Health Sciences, Liverpool Hope University, Liverpool, L16 9JD UK; 6 College of Life and Environmental Sciences, University of Exeter, Exeter, EX1 2LU UK; 7 EPSRC Centre for Predictive Modelling in Healthcare, University of Exeter, Exeter, EX4 4QJ UK

## Abstract

Children with developmental coordination disorder (DCD) struggle with the acquisition of coordinated motor skills. This paper adopts a dynamical systems perspective to assess how individual coordination solutions might emerge following an intervention that trained accurate gaze control in a throw and catch task. Kinematic data were collected from six upper body sensors from twenty-one children with DCD, using a 3D motion analysis system, before and after a 4-week training intervention. Covariance matrices between kinematic measures were computed and distances between pairs of covariance matrices calculated using Riemannian geometry. Multidimensional scaling was then used to analyse differences between coordination patterns. The gaze trained group revealed significantly higher total coordination (sum of all the pairwise covariances) following training than a technique-trained control group. While the increase in total coordination also significantly predicted improvement in task performance, the distinct post-intervention coordination patterns for the gaze trained group were not consistent. Additionally, the gaze trained group revealed individual coordination patterns for successful catch attempts that were different from all the coordination patterns before training, whereas the control group did not. Taken together, the results of this interdisciplinary study illustrate how gaze training may encourage the emergence of coordination via self-organization in children with DCD.

## Introduction

Children with developmental coordination disorder (DCD) have significant difficulty in acquiring and executing the essential, coordinated motor skills involved in self-care (e.g., dressing), recreational activities (e.g., ball skills), and academic performance (e.g., handwriting) compared to their typically developing counterparts^1^. DCD is estimated to affect around 6% of children^1^ and can have a significant impact on their socio-emotional wellbeing^2^ and future health status^3^. As such, there is a need for carefully designed and executed randomised control trials (RCT) to investigate the efficacy of interventions for children with DCD^4,5^. Additionally, given that children with DCD represent a very heterogeneous population; in terms of the range and variability of impairment, and the influence of co-occurring disorders, it is important that RCT outcome measures are sensitive to subtle and individual changes in coordination^6^. The current paper applies recently proposed statistical learning techniques to explore how children with DCD might find individualised, self-organising movement solutions following a group-based training intervention.

The intervention itself is grounded in research that has demonstrated that the quiet eye (QE)^7^ - an objective measure of visuomotor control in targeting and interception tasks - can be trained, with significant benefits for performance^8,9^. Wilson *et al*. were first to determine that the QE mediated performance differences between children of varying motor coordination abilities in a throw and catch task^10^. Highly proficient children revealed longer QE pursuit tracking durations – locating the ball more quickly and tracking it for longer - prior to more accurate catch attempts. This finding was not wholly surprising, as a body of evidence has linked DCD to significant impairments in general visuomotor control and the processing of task-relevant, visual information^11^; the ability to use predictive information to guide action^12^; and the pursuit tracking of objects^13^. However, particular strengths of the study^10^ were the use of a ‘real world’ throw and catch task that represents the building blocks for sport and playground games, and the collection of eye movement videos (from mobile eye trackers) of expert performers that could be used as feed-forward models for subsequent training interventions. Specifically, QE training videos were created that showed the expert eye movements from a first-person perspective alongside an auditory commentary that identified the key targets to be attended (see^14^ supplementary files, or http://see2learn.co.uk/videos/ for example videos).

Two separate RCTs subsequently showed that while children with DCD do have impairments in visual control – as evidenced by later and shorter QE durations on the incoming ball - this could be improved via QE training. Importantly, these improvements in gaze control (longer QE durations) also translated into performance improvements^14,15^. In comparison, a control group who received typical movement-focused video instructions (Technical Training; TT), revealed no improvement in QE or catching technique after training. The authors concluded that QE training served to improve the attentional control of these children, providing earlier information with which to prepare the interceptive catch attempt.

While group-based changes in measures of QE and performance quality were evident in both these studies, examining the movement patterns underpinning an improvement in performance provides important information from the perspective of motor control in DCD. Specifically, identifying if there were common or unique characteristics in technique change would help inform knowledge of how movement coordination emerges, and what training might be useful to underpin learning. Indeed, it is possible that children found different motor solutions to the catching problem. It has been suggested that by focusing on an external target, QE training allows the body to self-organise to meet the end point goal of getting the hands into position at the right time and place to make catching possible^15,16^. Research to date has not been able to test this hypothesis for both methodological and theoretical reasons. First, the focus in RCT studies is the detection of common improvements in the treatment group compared to the control group. Any variability in response to treatment is seen as a limitation of the generalizability of the intervention, as opposed to potential individualised solutions to the problem. This limitation is in turn related to the fact that few studies take a dynamical systems perspective to the self-organisation of movement under constraints^6,17–19^.

The dynamical systems perspective has its roots in biological systems theory and contends that the timing and coordination of movement are emergent properties of the individual physical system in its interaction with the environment^17,18^. Specifically, according to Newell’s^17^ model of constraints, movement coordination emerges as a consequence of the interaction of organismic, environmental and task constraints on the system. Optimal patterns of coordination and control therefore emerge from the unique confluence of constraints impinging on individual neuro-musculoskeletal systems through a process referred to as ‘self-organizing optimality’^17–20^. As such, a key principle of a dynamical systems approach to skill acquisition is that there is no single optimal technique for a goal-directed action (like catching a ball)^21^, which may be further exaggerated in the heterogeneous population of children with DCD^6^. While *end point* variability may be evidence of poor task performance, *coordinative* variability is associated with multiple ways of achieving the task goal via exploration of the perceptual-motor space^22,23^.

This dynamical systems perspective is under-represented in the DCD literature^6^ and has implications for how research into DCD is conducted. First, it calls into question how useful comparisons of endpoint variability between groups of typically developing (TD) children and children with DCD may be for understanding the particular constraints acting on an individual^6,24^. Recent reviews have lamented that while such comparisons are plentiful, they do not address either the ‘developmental’ or ‘coordination’ aspects of the disorder^6,24,25^. It is therefore important for research to assess intra-individual changes in coordination over time. The current study answers these calls by examining how children with DCD might learn to adopt different coordination solutions following a four-week intervention designed to improve throw and catch performance. Specifically, to measure the complexity inherent in biological systems, we applied methodology inspired by Kerkman *et al*.^26^ and used tools routinely applied in neuroscience and data mining^27–29^. Namely, we quantified coordination patterns using covariance matrices and Riemannian geometry and visualised the relationships between the patterns using multidimensional scaling.

The current study therefore has two main aims: (1) to provide additional support for a novel intervention^14^ and (2) to examine the emergence of coordination from a dynamical systems perspective, using novel measures. We have previously reported a training advantage for this intervention in terms of a subjective rating of ‘end point’ performance quality^14^ so wanted to examine if mathematically derived coordination measures would reveal similar group differences. Specifically, we hypothesized that (1) the QET participants would reveal increased coordination (movements occurring concurrently rather than subsequently), as evidenced by increased covariance of the kinematic measures following training, compared to their TT counterparts. Additionally, if the QE training intervention supported the creation of self-organizing solutions, we would hypothesize that (2) there would be high variability in the post training coordination patterns between and within individuals.

## Results

We first checked if covariances between pairs of kinematic measures increased after the intervention. We chose to use covariances rather than correlations, because they capture not only coordination between the measures but also inform us about the range of motion. In the QE trained group, we found a significant increase in absolute values of covariance (medians over trials) in 10 out of the 15 pairs of measures, while there was no such increase in the technique-trained (TT) control group (see Table 1). We interpret an increase in the absolute value of covariance as an increase in coordination; in practice it might for example mean that left and right arm were moving together or that movement of an arm was ‘smoother’ (e.g., elbow and shoulder were moving concurrently).

**Table 1:** Differences of median pair-wise absolute covariance values between the kinematic measures in the gaze (QET) and technique (TT) trained groups.

Pair of measures	QET	FDR	TT	FDR
1: Left elbow flexion,	**216.6**	**0.0342**	231.7	0.7341
2: Right elbow flexion				
1: Left elbow flexion,	**111.7**	**0.048**	0.7	0.7935
3: Left shoulder total flexion				
1: Left elbow flexion,	104.7	0.2789	−37.6	0.8513
4: Right shoulder total flexion				
1: Left elbow flexion,	**220.9**	**0.0386**	26.1	0.7935
5: Left shoulder flexion				
1: Left elbow flexion,	55.8	0.2854	37.8	0.7341
6: Right shoulder flexion				
2: Right elbow flexion,	**114.2**	**0.0423**	−20.3	0.7935
3: Left shoulder total flexion				
2: Right elbow flexion,	**131.9**	**0.0480**	57.6	0.7341
4: Right shoulder total flexion				
2: Right elbow flexion,	120.4	0.1162	42.6	0.7341
5: Left shoulder flexion				
2: Right elbow flexion,	115.2	0.1104	132	0.7043
6: Right shoulder flexion				
3: Left shoulder total flexion,	**159.2**	**0.0386**	17.6	0.7935
4: Right shoulder total flexion				
3: Left shoulder total flexion,	**287.4**	**0.0386**	45.6	0.7935
5: Left shoulder flexion				
3: Left shoulder total flexion,	**142.4**	**0.0423**	−18.2	0.8513
6: Right shoulder flexion				
4: Right shoulder total flexion,	**189.6**	**0.0165**	43.2	0.7935
5: Left shoulder flexion				
4: Right shoulder total flexion,	175.8	0.1575	37.5	0.7935
6: Right shoulder flexion				
5: Left shoulder flexion,	**160.9**	**0.039**	−144.9	0.8513
6: Right shoulder flexion				

Statistical test: one-sided Mann-Whitney-Wilcoxon test with Benjamini Hochberg false discovery ratio (FDR) correction for 15 tests in each group. In bold FDR < 0.05.

**Figure 1:**
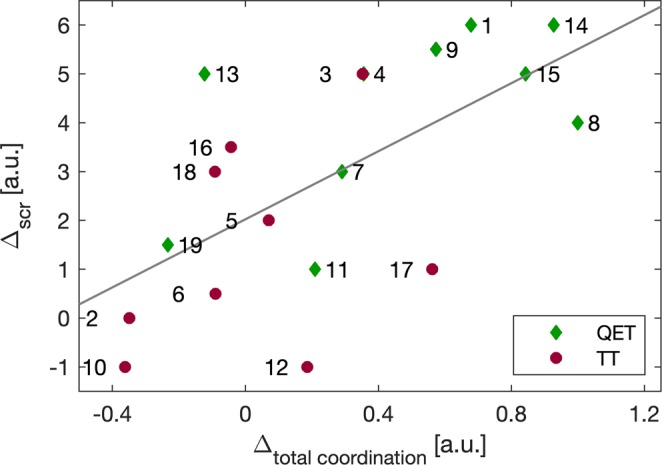
Plot of the change in total coordination (normalized), ∆_*TOTAL COORIDNATION*_, versus ∆_*SCR*_, the differences between the medians of catching scores of the participants (Pearson’s correlation coefficient R = 0.6279, p = 0.004). Green diamonds and red brown dots represent participants in QET and TT groups respectively and the grey line represents the regression line, y = 3.4834*x* + 2.0215.

We then checked to what extent change in the *total* coordination - defined as sum of absolute values of medians (over trials) of covariances between the 15 pairs of the 6 kinematic measures - explained improvement in catching performance. Figure 1 depicts the relationship between post-intervention change in total coordination, ∆_*TOTAL COORDINATION*_, for each participant against their change in catching score, ∆_SCR_. The change in total coordination predicted 39% of the variance in change of catching performance (Pearson’s correlation, *p* = 0.004); the greater the total coordination, the greater the improvement in catching performance. Overall, the QE trained group revealed an increase in total coordination after training; median ∆_*TOTAL COORDINATION*_ = 2463.2, whereas the total coordination of the technique-trained control group decreased; median ∆_*TOTAL COORDINATION*_ = −230.1, (p = 0.0175 one-sided Mann-Whitney-Wilcoxon test).

However, this basic analysis of the total coordination only provides a group-level understanding. To analyse changes in individual coordination *patterns*, we computed Riemannian distance between all the pairs of covariance matrices for all participants in all baseline and retention trials. We then used multidimensional scaling (MDS) to represent the matrices as points in an abstract geometric space given by two first principal dimension of the MDS. The two first principal dimensions represented 78% of the relations encoded in the raw Riemannian distances, meaning that they are suitable for visualization and analysis of the data^29^.

**Figure 2:**
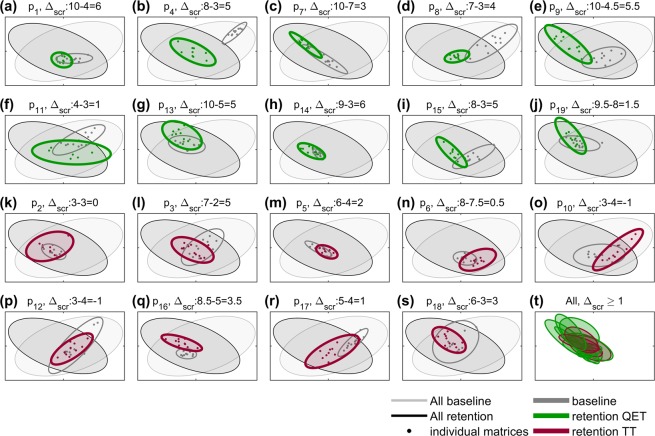
Plot of the changes in coordination patterns of individual participants, illustrated with two principal dimensions of multidimensional scaling. (**a**–**j**) Data for individual QET participants (green), (**k**–**s**) data for individual TT participants (brown-red). Small dots represent individual covariance matrices of each participant (grey – baseline, colour – retention); the dots are encircled by ellipses that represents 0.7 of the mass of fitted bivariate normal distribution. To show how individual participants compare with all others, the two large ellipses indicate the entire baseline (grey) and the entire retention (black) data; they represent 0.8 of the mass of fitted bivariate normal distribution. (**t**) Shows overlap between ellipses representing the covariance matrices of the retention trials in which on average participants achieved improvement ∆_SCR_ ≥ 1. Title shows participant’s identifier, median retention catching score, median baseline catching score and ∆_SCR_.

Figure 2 shows intra- and inter-personal variability. Points corresponding to all of the participants’ trials in a given condition form a cluster in the MDS space that we encircled with an ellipse representing a bivariate normal distribution fitted to the cluster of points on the plane (see^30^ for details of methodology for computing the ellipse). Interestingly, the QET participants showed a greater change in the coordination patterns than the TT participants but the nature of each change was individual specific. To measure the change in coordination patterns, we computed overlap ω between baseline and retention ellipses, with ω = 0 meaning that the ellipses did not overlap at all (i.e. the movements are completely different), and ω = 1 reflecting complete overlap (i.e. the movements did not change); median ω_QET_ = 0.2, median ω_TT_ = 0.31 (p = 0.0564 one-sided Mann-Whitney-Wilcoxon test).

It is also evident that the variability of the coordination pattern does not change in a consistent way. For example, Fig. 2(a) or (d) show less variability after QE training (the green ellipse is smaller than the grey), while Fig. 2(b) or (h) show higher variability after QE training (the green ellipse is larger than the grey). In order to further examine variability in coordination, we attempted to control for performance. First, we compared the retention test coordination patterns of all participants who improved performance after training (i.e. with *∆*_*SCR*_ ≥ 1). Figure 2(t) shows that while there is some similarity between these coordination patterns - there is a large degree of overlap between ellipses - the overlap is not complete, meaning that there are inter-personal differences.

**Figure 3:**
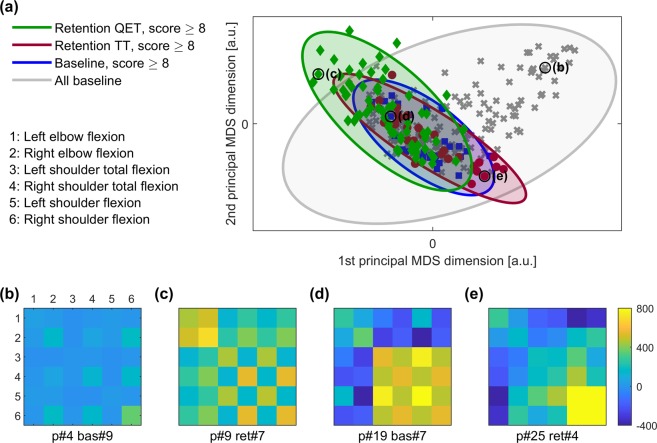
Plot of the changes in coordination patterns of trials in which participants achieved scores ≥ 8, illustrated with two principal dimensions of the multidimensional scaling. (**a**) Markers represent individual covariance matrices from all the baseline trials (grey crosses), 29 baseline trials with score ≥ 8 (blue squares), 30 retention trials from TT group with score ≥ 8 (red-brown circles), 66 retention trials from QET group with score ≥ 8 (red circles). The markers are encircled by ellipses that represents 0.7 of the mass of fitted bivariate normal distribution. The large grey ellipse encircles all the baseline markers and represents 0.8 of the mass of fitted bivariate normal distribution. (**b**–**e**) are representative examples of the covariance matrices; values in the matrices are colour coded. Markers in panel (**a**) that correspond to these exemplar matrices are indicated with a black circle and corresponding label.

To further investigate variability in the coordination patterns underpinning successful trial performance, we analysed covariance matrices from trials where participants achieved a catching score of 8 or higher (all trials where the ball was caught, even if not cleanly caught on the first attempt – see Methods). There were 29 such trials in the baseline condition, 30 among the retention trials of the TT group and 66 among the retention trials of the QET group. Figure 3(a) shows that there were a number of successful coordination patterns from the retention trials of the QET group (green diamonds) that were not similar to any of the baseline trials (11 green diamonds are outside of the big grey ellipse). It also shows that the successful coordination patterns from the retention trials of the TT group (red-brown circles) were always similar to the successful coordination patterns from the baseline trials (blue squares). Overall, this demonstrates that gaze training allowed for self-organization and emergence of individual coordination patterns that were different from all the movements in the baseline trials.

## Discussion

The main aim of this study was to investigate a dynamical systems approach to examining changes in coordination following training of children with DCD. This approach considers the role of self-organization under multiple constraints present at various levels within the child-task-environment interaction^6^. Both experimental and robotics fields have identified that an epistemological shift towards understanding the dynamics of a system within constraints - where redundancy and variability of the system are used to satisfy collective dynamics - may be mathematically, theoretically, and practically more fruitful^31^. It may also be particularly relevant for the study of children with DCD, who are a heterogeneous population in themselves^6^. The current study therefore addresses recent calls^6,24,25^ for this approach to be applied to better understand the coordination deficits inherent in DCD. Additionally, a dynamical systems approach enables us to explore the hypothesis that gaze (quiet eye) training might guide children with DCD to find individual coordination solutions via exploration of the perceptual-motor space, rather than creating a single ‘optimal’ pattern of coordination.

Our findings were supportive of both hypotheses. First, we found that the QE trained group had significantly higher coordination (as indexed by covariance values between the pairs of kinematic measures) than the technique-trained control group (Table 1) and that change in total coordination predicted 39% of the variance in change of catching performance (Fig. 1). In this way we validated our measure of coordination against a measure of end point performance and supported previous research claiming benefits of QE training for children with DCD^14,15^. Second, we showed that this group performance benefit occurred with significant intra- and inter person variability in the coordination patterns, as predicted (Fig. 2). In other words, while the coordination patterns of the QET participants changed more than their TT counterparts, this was not in a consistent manner (Fig. 2(a–j)). Indeed, we showed that the coordination patterns of some QET participants became more variable after training, while others became less variable.

Even when we controlled for performance, individual differences in coordination were evident. First, we compared the post-training coordination patterns of all participants who improved their catching performance from baseline to retention. While there was a large degree of overlap between ellipses (i.e. coordination patterns were similar), individual variability meant that this overlap was not complete (see Fig. 2(t)). Second, we compared coordination patterns for all trials in which participants managed to catch the ball. Again, there was evidence of individual variability in coordination patterns despite all reflecting successful (end point) performance (Fig. 3). Importantly - and in support of our second hypothesis - QE training allowed for self-organization and emergence of individual coordination patterns that were different from all patterns in the baseline trials. The successful coordination patterns from the retention trials of the TT group on the other hand, were always similar to those from baseline trials (Fig. 3).

The overall message is that while higher levels of covariance may be related to better performance, the distinct patterns of this coordination emerged in an individualised manner, depending on the unique constraints impinging on individual neuro-musculoskeletal systems^17^. Within this framework, individualized solutions are bound to exist as the specific environmental, individual, and task constraints will vary on a case-by-case (and throw by throw) basis^32^. As such, these findings support the benefit of developing a dynamical systems model of coordination in DCD and its applicability for further investigation of therapeutic training responses at the level of an individual. From this perspective, variability in movement systems is omnipresent and unavoidable due to the distinct constraints that shape each individual’s behaviour. Rather than being seen as something to limit, variability in movement systems may help individuals adapt to the unique constraints (personal, task and environmental) impinging on them across different timescales^17,33^.

The consideration of timescale is important when we consider the ‘developmental’ aspect of DCD. The pattern of coordination and control produced by the neuromusculoskeletal system is only optimised in relation to the immediately imposed constraints. Since the constraints imposed on an individual dynamical movement system (in this case a child with DCD) fluctuate continuously over time, the emergent pattern of coordination and control for any given motor activity will also change accordingly^33^. This is why research like the current study is important – in that it considers both the ‘developmental’ (change over time) and ‘coordination’ (interacting dynamics of the movement patterns) elements of DCD^6,24,25^. The ‘disorder’ element of DCD is also considered in this approach, in that it is recognised that while coordination patterns may be momentarily self-optimised, performance may still be lower than that of their typically developing peers^34^.

Interventions therefore need to be able to provide benefits within this changing landscape and thus the current study has some important implications for therapy. It is important to understand the different compensatory strategies that children with DCD might adopt to find adequate – even if not optimal – solutions to motor problems, based on their unique constraints, if interventions are to be personalized for their recipients. If initial system conditions are different – and constantly changing – it is unlikely that standardized interventions designed to coach specific movement solutions will be appropriate for children with DCD. A dynamical systems approach to understanding the deficits in perceptual-motor coordination of children with DCD provides a useful framework for designing personalized interventions that consider the individual constraints under which a child operates^35–37^.

This paper demonstrates one of the first interventions to show that allowing for self-organisation is actually better than prescribing technique in a DCD population. Additionally, while the gaze instructions themselves were standardised, they appeared to provide such a launchpad for self-organisation. We propose that this is because instructions relate to the perception component (quiet eye) of the perception-action coupling, rather than the action (specific technique cues) component. Key task relevant information is prioritised, but the means to achieving the task goal is via exploration of the perceptual-motor space^37^. These findings also support previous research that suggests that QE training provides a more implicit form of learning, relying less on conscious motor control than when providing explicit technical training instructions^38^. Future research should seek to employ similar mathematical approaches as adopted in the current study to explore the coordination between ongoing gaze behaviour (e.g., QE) and kinematic measures to further develop QE theory^39^.

From a practical, therapeutic perspective, the data also suggest that while children with DCD may reveal greater spatial variability than their TD counterparts^34^, reducing variability should not be the sole focus of any intervention designed to improve movement outcomes for this group. Therapeutic sessions may then be best structured around guiding DCD children to utilize task-specific sources of information rather than attempting to guide movement effectiveness directly, particularly through explicit instruction^40^. Support for this contention is also underlined by previous research that has suggested that the deficits associated with DCD are linked with a child’s persistence with ineffective strategies rather than a generalized inability to learn motor movements^41^. We have now shown that – when guided appropriately via targeted videos - children with DCD can learn to optimise effective gaze control^14,15^ and, in doing so, become more coordinated. While^14^ provided some indication (from parental reports) that QE training had a positive impact on subsequent sporting participation, future research needs to explore whether this newly learned strategy can transfer to other skills, and impact on ongoing and extended physical activity and social integration^2,3^.

To conclude, the current study reveals the potential advantages of applying data science techniques to assess trial by trial variability in movement patterns for intervention studies involving clinical groups; in this case, children with DCD. We provide additional support for the efficacy of gaze training interventions, as evidenced by increased coordination between kinematic measures following training. Despite these group differences in coordination, there was considerable variability in the individual coordination patterns within and between groups (and trials). Evidence was therefore found for a self-organization approach, where improved post-training movement coordination emerged from the unique constraints impinging on individuals. These findings suggest that interventions that train the perception element of a visually guided movement task (e.g., QE Training) do not enforce a particular, rigid movement solution, but instead provide perceptual information by which individuals develop their own individual solutions.

## Methods

### Data Collection

**Figure 4:**
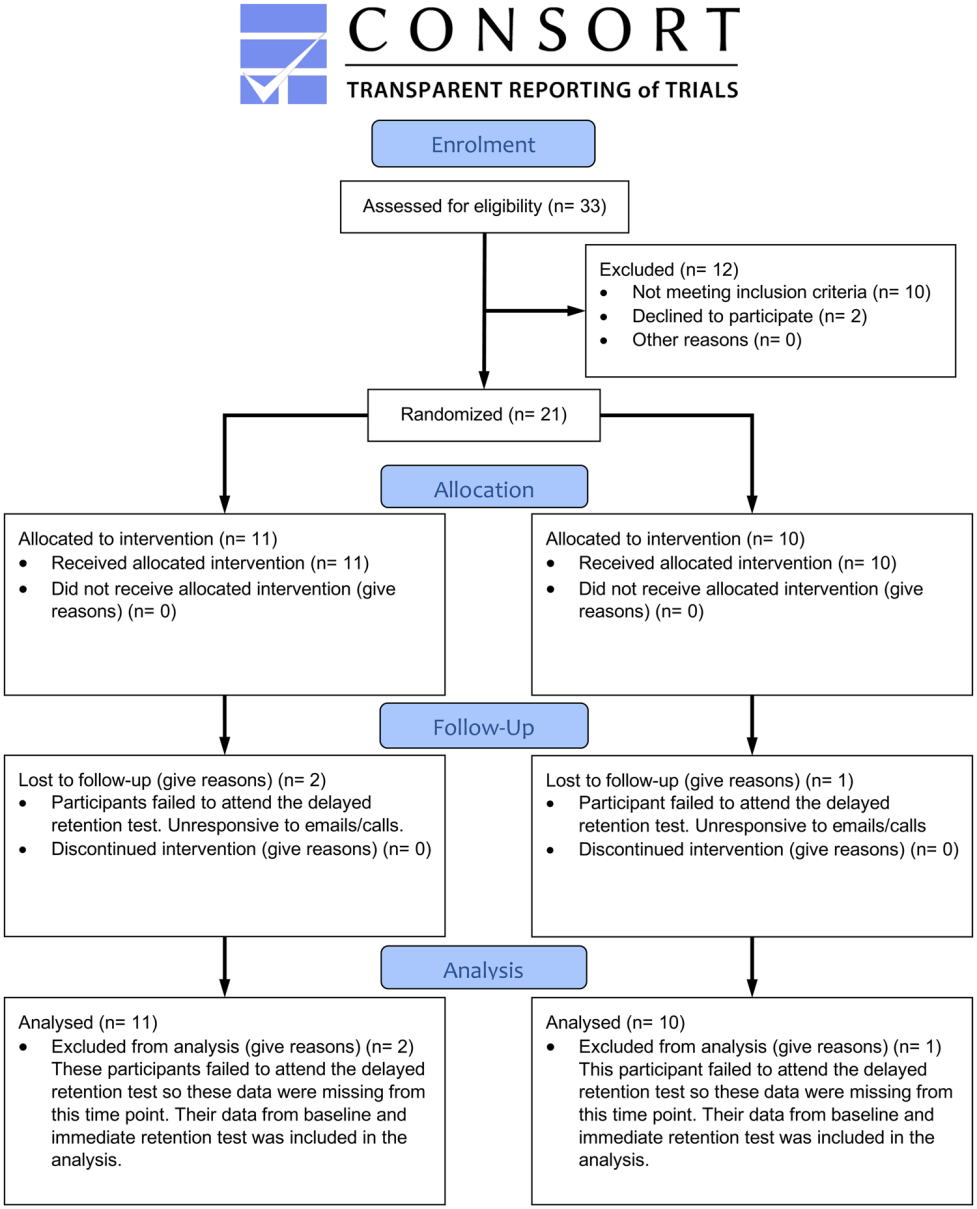
Consort 2010 Flow diagram for participant selection into the trial.

The participants were twenty-one children (aged 7–10) who scored below the 5th percentile on the Movement Assessment Battery for Children-2 (MABC-2)^42^. In line with the additional DSM-5 criteria for DCD^1^, all children were classified as of ‘normal’ intelligence based on their teacher/parent reports and scored below the cut-off (98^th^ percentile) on parental reports of the Attention Deficit/Hyperactivity Disorder (ADHD) Rating Scale-VI^43^. NHS ethical approval (15/NW/0279) was granted by the RES Committee North West-Greater Manchester South, before any testing was carried out, and parents and children provided written informed consent before taking part. All methods were carried out in accordance with these ethical guidelines and regulations. Figure 4 shows a CONSORT flow diagram outlining participant recruitment and analysis (The CONSORT checklist is available as supplementary data in^14^). The trial was registered on the 19^th^ September 2016 (see https://clinicaltrials.gov/ct2/show/NCT02904980) and more detail on the full trial protocol is provided in Supplementary Methods.

Participants performed 50 trials of the throw and catch task from the MABC-2 at three time points: before (baseline) and after training (retention), and at a 6-week delayed retention test. The throw and catch task required children to throw a ball against a wall two meters away and try to catch it on its return – without letting the ball bounce - using both hands^42^. Based on baseline measures, participants were pseudo-randomly divided into either Quiet Eye training (QET) (8 male 3 female, mean age of 8.6 years (*SD* = 1.04); mean MABC-2% of 2.6 (*SD* = 2.09); mean ADHD% of 87.9 (*SD* = 17.1)) or technical training (TT) groups (7 male 3 female, mean age of 8.6 years (*SD* = 1.84); mean MABC-2% of 1.9 (*SD* = 2.19); mean ADHD% of 90.5 (*SD* = 14.7)).

Training consisted of a 4-week group therapy intervention, involving a combination of observational learning via videos, and team games/exercises designed to reinforce the learning points (see 10.1371/journal.pone.0171782.t002 and supplementary data for details). Week 1 of training focused on accurate throwing, week 2 on effective catching, week 3 on linking the throw and catch, and week 4 served as a summary week in which children selected their favourite activities from the previous three weeks. The TT group were given movement-related instructions via video, relating to the throw and catch phases; specifically, by training them to adopt a smooth arm swing during the throw, and to ready themselves and use soft hands to catch. The QET group’s video instructed them how to adopt expert-like gaze control; specifically, by training them to fixate a target location on the wall prior to the throw and to track the ball prior to the catch (videos available as supplementary files in^14^). The training games (e.g., throwing to targets, catching on the move) were the same for each group but the instructions provided related to the instructions provided in the training videos.

### Data Acquisition

A 3D motion analysis system (MyoMotion Research Pro, Noraxon Inc., Scottsdale, AZ, USA) was used to collect kinematic data from six upper limb 3D inertial motion capture sensors fitted according to the Noraxon standard manual (Noraxon Inc., Scottsdale, AZ, USA). Two sensors were located on each upper and lower arm, and one sensor on both the pelvis and cervical spine, allowing shoulder and elbow angles and range of motion (ROM, degrees) to be determined for both arms (sampling frequency of 100 Hz). In total, six kinematic measures were computed by the Noraxon software; left and right elbow flexion, shoulder total flexion (taking into account flexion and abduction around the shoulder), and shoulder flexion. Calibration was performed in the standing position to define the 0° of ROM.

Kinematic data were only collected for the first 10 of the 50 trials at each time point, as (1) pilot testing showed that participants frequently struggled to not interfere with the sensors and the eye tracker when worn for too long, and (2) our previous studies had only used 10 trials in each condition^10,14,15^. Due to calibration problems, we only had complete kinematic data for 19 participants (9 TT and 10 QET) and in this analysis we focus on pre-post intervention data (i.e. baseline to immediate retention) in order to access immediate individual differences due to training.

### Data Processing and Analysis

Data were recorded as time series continuously during the task, so each time series contains ten task trials for each participant. First, we divided the time series into trials (time series segments) by detecting the throwing and catching periods. In our analysis, we focus on the “catching period” (from ball release to catch), which is expected to have highest variability between participants and can be more closely compared to a measure of catching performance.

#### Coordination Patterns Evaluation

**Figure 5:**
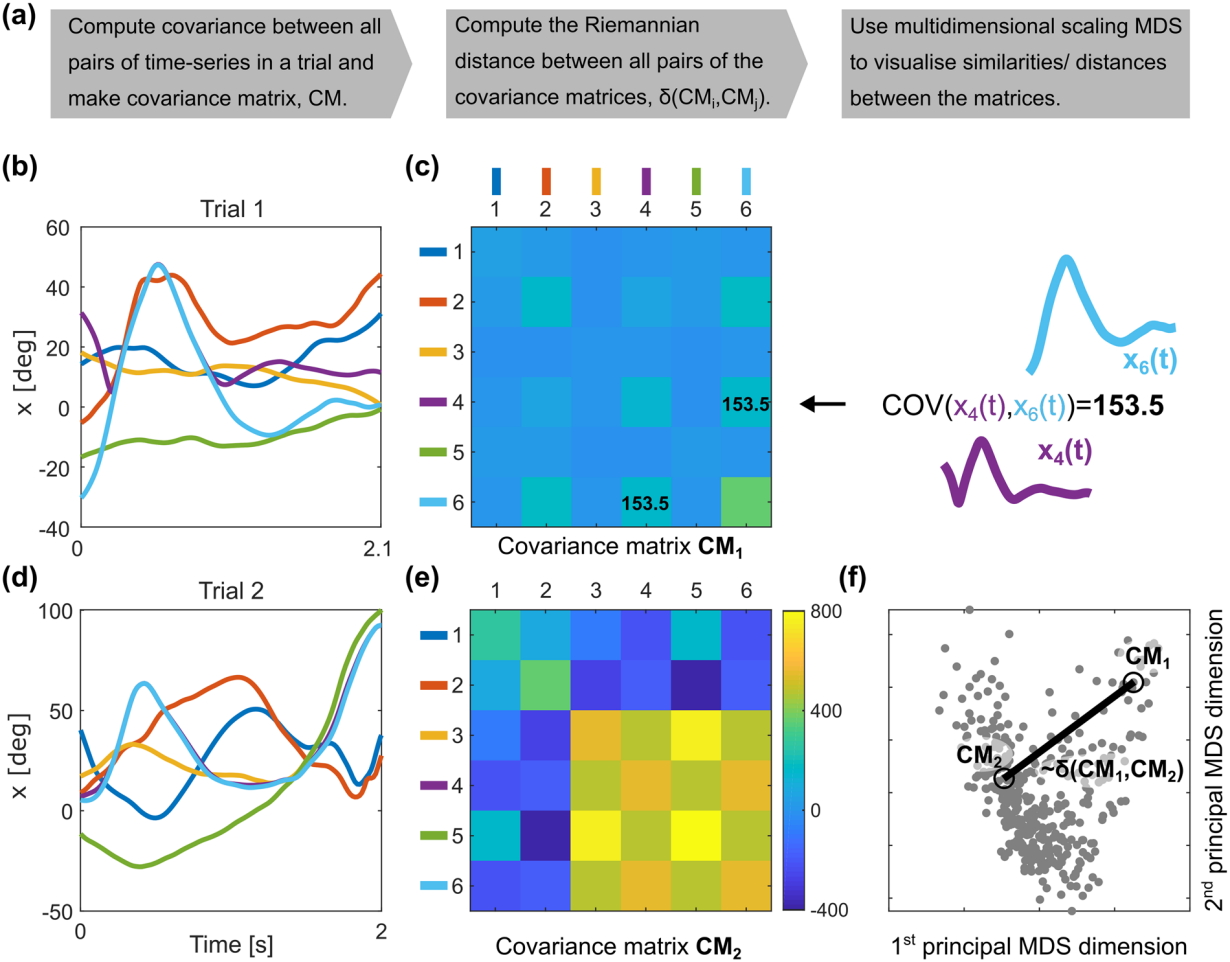
(**a**) Data processing and analysis pipeline. (**b**,**d**) Time series of kinematic measures: 1: Left elbow flexion, 2: Right elbow flexion, 3: Left shoulder total flexion, 4: Right shoulder total flexion, 5: Left shoulder flexion, 6: Right shoulder flexion. (**c**,**e**) Their covariance matrices CM_1_ and CM_2_, covariance values are colour coded. (**f**) Two first principal dimensions of the multidimensional scaling. Each dot represents a covariance matrix from a single trial. Dots representing covariance matrices CM_1_ and CM_2_ are indicated with black circles. Distances between the dots on the plane of the principal MDS dimensions (black line) are an approximation of the Riemannian distance, δ(CM_1_,CM_2_), between the covariance matrices.

Figure 5(a) provides a summary of the three-step approach adopted to compare coordination before and after training. To consider the temporal relationships (coordination) between kinematic measures, we first computed the covariance between them. The covariance between two data sets *A* and *D* is defined as:

$$cov(A,D)=\frac{1}{N-1}\sum _{i=1}^{N}({A}_{i}-{\mu }_{A})({D}_{i}-{\mu }_{D})$$

where *μ*_*A*_ and *μ*_*D*_ are the mean values of the sets *A* and *D* respectively, and *N* is the number of samples in the sets. Figure 5(b,d) shows exemplar time series data of the catching period of two different trials. Covariances between pairs of the time series were next saved in a matrix; Fig. 5(c,e).

Second, after computing the covariance matrices for all trials for all participants, we then estimated the distance between these covariance matrices by applying a Riemannian geometry approach^27,28^. Riemannian geometry allows us to analyze data that lie in a curved space, where we can no longer apply Euclidian space operators, which is the case for covariance matrices^27^. The Riemannian distance between two covariance matrices *CM*_1_ and *CM*_2_ is given by:

$$\delta (C{M}_{1},C{M}_{2})=\sqrt{\sum _{n=1}^{N}lo{g}^{2}{\lambda }_{n}}$$

where *λ*_*n*_ are the N eigenvalues of matrix $C{M}_{1}^{-1/2}C{M}_{2}C{{M}_{1}}^{-1/2}$ (or equivalently $C{M}_{1}^{-1}C{M}_{2}$)^28^.

Third, once the distances between all the pairs of the covariance matrices were calculated, we used Multidimensional Scaling (MDS) to analyse and visualise the differences and similarities between coordination patterns. MDS is a data analysis technique widely applied to visualise the similarities/ differences between data sets^29^ (see^30^ for an example in movement analysis). MDS allows us to represent the covariance matrix of each participant as a dot in an abstract geometric space. For visualisation purposes we used the first two (i.e. most significant) dimensions of this abstract space. Figure 5(f) shows a visualisation of all the covariance matrices from all trials using two first principal dimension of the MDS of all the Riemannian distances between pairs of the covariance matrices from the current study.

#### Catching Score

The catching performance scale^14,15^ was used to assess the quality of each attempted catch on an 11-point scale, between ‘0’ (Makes no move towards the ball as it comes back) and ‘10’ (The catch is made exclusively with the palms and fingers). The assessment was made by a researcher - blinded to training group status – from video recordings taken of all catching attempts. Each number on the scale has an associated description (e.g., ‘6’ – Ball hits body and is trapped with arms but not hands – see https://journals.plos.org/plosone/article?id=10.1371/journal.pone.0171782). A mean value was then computed for baseline and retention conditions and used in subsequent analyses. We have previously reported a significant difference in training effect for catching score for the participants of this trial^14^: The control group did not significantly improve (Bonferroni-corrected *p* = 0.028) from baseline (M = 3.73, *SD* = 2.02) to retention (M = 5.45, *SD* = 2.30), whereas the QE Trained group did (M = 4.10, *SD* = 1.58, to M = 6.54, *SD* = 2.06; Bonferroni-corrected *p* < 0.001).

#### Statistical and Computational Methods

To test statistical significance of our findings we used non-parametric Mann-Whitney-Wilcoxon test as implemented in Matlab with command ranksum^44^. Additionally, where appropriate we control for multiple comparison using Benjamini Hochberg false discovery ratio method^45^ as implemented in Matlab with command mafdr (…,‘BHFDR’, 1). To assess correlations we used Pearson R coefficient of linear dependence [R, p] = corr(x,y, ‘type’, ‘Pearson’). Covariance matrices and distances between them were computed in Matlab using cov and distance_riemann, commands respectively. The function distance_riemann computes the Riemannian distances, and can be found in a freely available toolbox called *Covariance Toolbox (*https://github.com/alexandrebarachant/covariancetoolbox*)*. The Matlab command for MDS is cmdscale.

Detailed methods and preliminary performance and qualitative data from this trial (ClinicalTrials.gov NCT02904980, 19th September 2016) were published in Wood *et al*. (2017) PLoS ONE 12(2): e0171782. doi:10.1371/journal. pone.0171782.

## Supplementary information

Supplementary Methods

## Data Availability

The datasets generated and analysed during the current study are available at the University of Exeter online repository; DOI:10.24378/exe.783.
