# Supplementary material for: Gaze training supports self-organization of movement coordination in children with developmental coordination disorder
Source: Sci Rep. 2019 Feb 8;9:1712. doi: 10.1038/s41598-018-38204-z (PMC6368583; doi:10.1038/s41598-018-38204-z)
Supplement: Supplementary file 1 — Supplementary Methods [file 41598_2018_38204_MOESM1_ESM.pdf]

Supplementary Methods for "Gaze training supports self-organization of movement coordination in children with developmental coordination disorder"

AUTHORS: Piotr Słowiński, Harun Baldemir, Greg Wood, Omid Alizadehkhaiyat, Ginny Coyles, Samuel Vine, Genevieve Williams, Krasimira Tsaneva-Atanasova, Mark Wilson

Trial Protocol - see https://clinicaltrials.gov/ct2/show/study/NCT02904980

Project Summary
The aim of this study was to integrate a gaze training intervention (i.e., quiet eye training; QET) that has been shown to improve the throwing and catching skill of children with Developmental Coordination Disorder (DCD), within an approach (i.e., group therapy) that might alleviate the psychosocial influence of these motor skill deficits. Twenty-one children (aged 7-11) with DCD completed throw and catch tests at baseline, one week following a four week training programme (retention) and a further six weeks after that (delayed retention). After baseline, participants were split into one of two groups that received one hour of training, weekly, for four weeks. Training first involved watching video-based instructions, with associated group-based practice activities to follow. The QET group videos highlighted the gaze footage of an expert model, whereas the control (technical training [TT]) group videos highlighted the limb movement of the expert model.
Performance across the three time periods was assessed by measuring (1) catch success rates, (2) catch performance quality (a rating of the quality of the catch attempt); (3) gaze metrics taken from a mobile gaze registration system; (4) kinematic and electromyographic measures of movement effectiveness. Finally, (5) parental feedback to questions about psychosocial and motor skills outcomes was collected at delayed feedback.
A previous report (https://journals.plos.org/plosone/article?id=10.1371/journal.pone.0171782#pone.0171782.s002) has discussed measures (1-3, 5) and the aim of the current report was to investigate changes in movement efficiency (4) and discuss these in relation to changes in movement effectiveness (2).

General Information

Title. The Effects of a Group-based Gaze Training Intervention for Children With Developmental Coordination Disorder.
ClinicalTrials.gov Identifier. NCT02904980, 19.09.2016.
Sponsor. Manchester Metropolitan University, Crewe, Cheshire, United Kingdom, CW1 5DU
Collaborators. Liverpool Hope University, University of Exeter, University of Calgary, The Waterloo Foundation.
Lead Researchers. Dr Greg Wood (Manchester Metropolitan University) and Prof Mark Wilson (University of Exeter).
Study Director. Prof Omid Alizadehkhaiyat (Liverpool Hope University)

Rationale & Background Information
Children with developmental coordination disorder (DCD) have significant difficulty in acquiring and executing the essential, coordinated motor skills involved in self-care (e.g., dressing), recreational activities (e.g., ball skills), and academic performance (e.g., handwriting) compared to their typically developing counterparts [1]. DCD is estimated to affect around 6% of children [1] and can have a significant impact on their socio-emotional

wellbeing [2] and future health status [3]. As such, there is a need for carefully designed and executed randomised control trials (RCT) to investigate the efficacy of interventions for children with DCD [4,5].

The intervention presented in the current manuscript is grounded in research that has demonstrated that the quiet eye (QE) [6] - an objective measure of visuomotor control in targeting and interception tasks - can be trained, with significant benefits for performance [7,8]. Wilson et al. were first to determine that the QE mediated performance differences between children of varying motor coordination abilities in a throw and catch task [9]. Highly proficient children revealed longer QE pursuit tracking durations – locating the ball more quickly and tracking it for longer - prior to more accurate catch attempts.

Two separate RCTs subsequently showed that while children with DCD do have impairments in visual control – as evidenced by later and shorter QE durations on the incoming ball - this could be improved via QE training. Importantly, these improvements in gaze control (longer QE durations) also translated into performance improvements [10,11]. In comparison, control groups who received typical movement-focused video instructions (Technical Training; TT), revealed no improvement in QE or catching technique after training. The authors concluded that QE training served to improve the attentional control of these children, providing earlier information with which to prepare the interceptive catch attempt.

The current study sought to further explore the advantages of QET using more sophisticated measures of coordination, such as muscular activity (EMG) and 3D kinematic analysis during the task. This approach will give us a greater understanding of exactly how the QET optimises throwing and catching and may also give a greater insight into the visuomotor difficulties typical in children with DCD. Additionally, rather than using a single, individual lab-based training session, participants took part in four, weekly group training sessions, aimed at exploring some of the social issues associated with DCD [2].

Study Goals and Objectives
The objective of this research was to determine the efficacy of a rigorous gaze training technique (quiet eye training) to improve the motor skill performance of children with Developmental Coordination Disorder (DCD) over that of traditionally taught techniques.

Study Design

Study Type. Interventional (Clinical Trial)
Actual Enrolment. 21 participants
Allocation. Randomized
Intervention Model. Parallel Assignment
Masking. Single (Participant)
Primary Purpose. Treatment
Official Title. A Randomised Controlled Trial of a Group-based, Gaze Training Intervention for Children With Developmental Coordination Disorder
Study Start Date. February 2015
Actual Primary Completion Date. September 2015
Actual Study Completion Date. September 2015

Participants
Twenty-one children aged 7–11 years old, were recruited from Alder Hey Children's Physiotherapy department, local DCD support groups, social media, and local occupational

therapy centres in the North West of England. Recruitment and follow-up took place between February 2015 and September 2015. All children scored below the 5th percentile on the Movement Assessment Battery for Children-2 (MABC-2) [12] carried out at the baseline testing phase. For more information on the demographic and clinical characteristics of the Quiet Eye Training (QET) and Technical Training (TT) groups, please see, https://doi.org/10.1371/journal.pone.0171782.t001.

Inclusion Criteria

- Prior diagnosis of Developmental Coordination Disorder or suspected to have DCD
- Scores below the 5th percentile on the MABC-2 (Movement Assessment Battery for Children-2)
- Be of normal intelligence (assessed through parent/teacher feedback)
- No neurological disorder
- Normal of corrected-to-normal vision

Exclusion Criteria:

- Score over 5th Percentile on the MABC-2
- Suffers from a neurological disorder
- Below average intelligence
- Any visual impairment

Assessment Measure

The throwing and catching task from the MABC-2 (8–10 year age bracket [12]) was used to assess performance and aid comparison with previous studies [9,10,11]. It requires participants to stand behind a line situated 2 metres from a wall, throw a tennis ball against the wall using an under-arm action, and catch it cleanly with two hands before it bounces. In line with MABC-2 instructions, the task was first explained to the participant and demonstrated once, before the participant took five practice attempts.

Apparatus

Each participant was fitted with an Applied Science Laboratories' Mobile Eye XG gaze registration system (ASL, Bedford, MA), measuring point of gaze at 30 Hz. A 30 Hz Digital SLR camera (Finepix S6500fd) was placed on a tripod 2 metres to the right of the throw line at shoulder height of the participant. This captured a side-on view (sagittal plane) of the participant's movements during the throw and catch action. Additionally, children were fitted with six upper limb 3D inertial motion capture sensors (two on each arm, one on the pelvis and one on the cervical spine) and 11 surface EMG electrodes to the upper and lower body muscles (Noraxon, USA).

Procedure

Testing was divided into baseline, training, retention and delayed retention phases.

Baseline. During the baseline phase, children attended the laboratory individually (with a parent / guardian), and following the completion of written consent, completed the MABC-2 protocol. The child was then fitted with an ASL gaze registration system and had EMG electrodes and kinematic markers attached to their arms and shoulders. Prior to completing the throwing and catching task, each participant was calibrated to the gaze registration system using nine locations on the wall to which they were required to throw the ball against. They then completed five blocks of 10 trials of the MABC-2 throw and catch task. Parents / guardians were asked to complete an information form about the child's

condition(s) and an ADHD checklist [13]. The baseline phase took approximately 60 minutes.

Retention. The procedure for the retention and delayed retention phases replicated the baseline phase; after having the gaze registration system and sensors fitted, participants completed five blocks of 10 trials of the MABC-2 throw and catch task. The retention phase occurred 1 week after the end of the training phase (week 6) and the delayed retention phase took place 6-weeks after this point. During the delayed-retention phase, parents completed a parental feedback questionnaire while their child completed the throwing and catching task procedure. All parents and children were blind to their group allocation.

Training. Participants were pseudo-randomly divided (to control for baseline performance differences) into two experimental groups based on this initial throwing and catching performance and entered the training phase of the study. A novel 4-week training protocol was developed in which participants attended weekly, group training sessions held at Liverpool Hope University Sports Hall. The training included a combination of videos and exercises targeted at explicitly teaching the children the required technique. Each training session took approximately 60mins and consisted of up to 15 children. At least two qualified coaches/researchers were present throughout all sessions. Week 1 of training focused on accurate throwing, week 2 on effective catching, week 3 on linking the throw and catch, and week 4 served as a summary week in which children selected their favourite games from the previous three weeks.

A week-by-week breakdown of the training activities and instructions for Quiet Eye Trained (QET) and Technically Trained (TT) groups (copied from https://doi.org/10.1371/journal.pone.0171782.t002).

Week	Activities	QET Instructions	TT Instructions
**Week 1 –Accurate Throwing**	• 20 warm up MABC-2 throw and catches<br>• Watch instructional video for throwing<br>• Target-related activities (throwing bean bags into buckets, throwing balls at cricket stumps, throwing at cut-out faces stuck on a wall).<br>• Started at short distances and increase distance based on individual success<br>• Competitive team games using the same tasks<br>• De-brief and reinforced instructions	Focus your eyes on the target and count to two before you start a smooth throwing action	Throw at a target using a smooth throwing action.
**Week 2 –Effective catching**	• 20 warm up MABC-2 throw and catches.<br>• Watch instructional video for catching<br>• Catching-related activities (catching large sponge balls and beanbags, catching with a bucket instead of their hands, catching while moving around).<br>• Varied distance and speed of the catch<br>• Competitive team games using the same tasks.<br>• De-brief and reinforced instruction	Keep your eye on the ball until it comes back into your cupped hands	Concentrate on the ball and cup your hands together.
**Week 3 –Linking throwing and catching**	• 20 warm up MABC-2 throw and catches<br>• Watch instructional video linking the throw and catch<br>• Throwing and catching tasks (throwing and catching between participants while walking around, throwing and catching a ball along a chain, rounders with a larger sponge ball where children hit the ball with their hands rather than a bat)	Questioning on previous instructions and instruction on combining coaching points together	Questioning on previous instructions and instruction on combining coaching points together.
**Week 4 –Throwing and catching competitive games**	• 20 warm up MABC-2 throw and catches<br>• Children chose their favourite games from the sessions and were prompted to remember the related coaching points	Questioning on previous instructions and reiteration of coaching points	Questioning on previous instructions and reiteration of coaching points

doi:10.1371/journal.pone.0171782.t002

Videos. At the beginning of each session, each group watched a brief instructional video showing the same expert model completing the throwing and catching task. Both videos provided a split screen of the model showing a side-on view of their movement and a first-person view taken from the eye tracker, showing the point of gaze while performing the task. The videos were edited to reinforce the different training instructions. The TT group videos highlighted the movement of the expert model whereas the QET group videos highlighted the gaze footage of the same expert model. Examples of these videos for week 2 (training the catch) can be found at; https://doi.org/10.1371/journal.pone.0171782.s003

Activities. After watching each video, participants were questioned regarding its content to check their understanding. Participants then completed 20 'warm-up' trials of the MABC-2 throwing and catching task in unison, while coaches reinforced the respective coaching instructions. Throughout the intervention both groups took part in exactly the same throwing, catching and related interception type activities but the instructions that were emphasised by the coaches were different for each group.

The TT group received technique-based instructions related to the mechanics of the skill, taken from a UK physical education resource [14]. The QET group received instructions that were related to controlling their eye movements so that they tracked the ball for longer (based on our previous findings [11]

The group games in week 1 focused on target related activities (throwing bean bags into buckets, throwing balls at cricket stumps, throwing balls at cut out faces stuck on the wall). In week 2, games focused on catching activities (catching large sponge balls and beanbags, catching with a bucket and catching while moving around). In week 3, games combined throwing and catching (e.g., throwing and catching while walking around or in a chain), whereas in week 4 children chose their favourite games from previous sessions.

References (of literature cited in preceding sections)

1. American Psychiatric Association. *Diagnostic and statistical manual of mental disorders (DSM-5).* (American Psychiatric Pub, 2013).
2. Chen, Y.W., Tseng, M.H., Hu, F.C. & Cermak, S.A. Psychosocial adjustment and attention in children with developmental coordination disorder using different motor tests. *Res. Dev. Disabil.* 30, 1367–1377 (2009). doi: 10.1016/j.ridd.2009.06.004.
3. Cairney, J. & Veldhuizen, S. Is developmental coordination disorder a fundamental cause of inactivity and poor health-related fitness in children? *Dev. Med. Child Neuro.* 55, 55–58 (2013). doi: 10.1111/dmcn.12308.
4. Miyahara, M., Hillier, S.L., Pridham, L. & Nakagawa, S. Task-oriented interventions for children with developmental coordination disorder. *Cochrane Db. Syst. Rev.* 7, doi.10.1002/14651858.CD010914.pub2 (2017).
5. Smits-Engelsman, B., *et al.* Evaluating the evidence for motor-based interventions in developmental coordination disorder: A systematic review and meta-analysis. *Res. Dev. Disabil.* 74, 72-102 (2018).
6. Vickers, J.N. Visual control when aiming at a far target. *J. Exp. Psychol. Hum. Percept. Perform.* 22, 342-354 (1996).
7. Lebeau J.-C. *et al.* Quiet eye and performance in sport: A meta-analysis. *J. Sport Exerc. Psychol.* 38, 441-457. doi: 10.1123/jsep.2015-0123 (2016).
8. Rienhoff, R., Tirp, J., Strauss, B., Baker, J. & Schorer, J. The 'quiet eye' and motor performance: A systematic review based on Newell's constraints-led model. *Sports Med.* 46, 589–603 (2016). doi: 10.1007/s40279-015-0442-4.

9. Wilson, M.R., Miles, C.A., Vine, S.J. & Vickers, J.N. Quiet eye distinguishes children of high and low motor coordination abilities. *Med. Sci. Sports Exerc.* 45, 1144-1151 (2013).
10. Wood, G. *et al.* A randomized controlled trial of a group- based gaze training intervention for children with developmental coordination disorder. *PLoS ONE* 12(2), e0171782. doi:10.1371/journal. pone.0171782 (2017).
11. Miles, C.A., Wood, G., Vine, S.J., Vickers, J.N. & Wilson, M.R. Quiet eye training facilitates visuomotor coordination in children with developmental coordination disorder. *Res. Dev. Disabil.* 40, 31-41 (2015).
12. Henderson, S.E., Sugden, D.A. & Barnett, A.L. *Movement Assessment Battery for Children-2: Movement ABC-2: Examiner's manual.* (London, Pearson, 2007).
13. DuPaul, G.J. *et al.* Parent ratings of attention-deficit/hyperactivity disorder symptoms: Factor structure and normative data. *J. Psychopathol. Behav. Assess.* 20, 83–102 (1998).
14. Bunker D, Hardy C, Smith B, Almond L. *Primary physical education: Implementing the national curriculum.* Cambridge: Cambridge University Press (1994).

Ethics and Safety Considerations

UK NHS ethical approval (15/NW/0279) was granted by the RES Committee North West—Greater Manchester South, before any testing was carried out, and parents and children provided written informed consent before taking part.

Each participant was asked to perform a number of throwing and catching activities. There was a minimal risk of fatigue during the assessments and training phases. To manage this, participants were encouraged to rest between blocks of trials and were informed that should they wish to have a break at any point then they were free to do so.
There was also a small risk of injury caused by tripping or falling or being contacted by a tennis ball during the training sessions. The area was be risk assessed and checked for trip hazards before the children arrived at each session and at least two adults supervised children at all times (including a qualified sports coach). The activities fell under the sports hall existing risk assessments, so no new risk assessments were required for the study. A trained first aider was on the premises at all times.

The set up and calibration of the equipment used to collect the dependent variables, although potentially time consuming, were non−invasive and had minimal associated risks.

A research problem in our previous studies was recruitment, but this was the first time we recruited directly from a hospital (Alder Hey, Children’s Physiotherpay UnitLiverpool), rather than specialist schools or help groups.

Recruitment. Upon registering their interest in the study, parents were sent an information pack that included the parent-specific information sheet and informed consent form. This will be signed by the parent and sent back to the research team. On the child's first visit to the lab a researcher went through the child's version of the information sheet and informed consent form.

Confidentiality. Data of the participant's movements and gaze behaviour were recorded on two separate recording devices. This was digitised onto a computer hard drive by a researcher and given a coded name that protected the participant's identity which were then used for all information relating to that participant. All personal data (both computer files,

film and paper) were destroyed on completion of the study. Anonymised data (used for subsequent analyses) were stored on password protected computers and are now available (see below).

Data Management

1. Data from Wood et al. (2017) can be located at:
   https://e-space.mmu.ac.uk/620164/
   Deposited (20th March 2018)

2. Data from additional mathematical analyses included in the current manuscript can be located at:
   DOI:10.24378/exe.783
   Deposited (6th November 2018)

Finance
This research was funded by grants from Liverpool Hope University (HEIF5 Programme 2011-2015) and The Waterloo Foundation (1119/1603). HEIF5 provided funding for a one year research associate position (for Dr C.A.L. Miles) to manage the trial. The Waterloo Foundation provided additional funds for Prof M.R. Wilson to assist in the project and for Dr H. Baldemir's time to perform the initial mathematic analysis reported in the current manuscript.
